# Supplementary material for: The role of extracellular matrix phosphorylation on energy dissipation in bone
Source: eLife. 2020 Dec 9;9:e58184. doi: 10.7554/eLife.58184 (PMC7746230; doi:10.7554/eLife.58184)
Supplement: Supplementary file 1. [file elife-58184-supp1.docx]

| Mean Energy | | | | |
| --- | --- | --- | --- | --- |
|  | **EDTA** | ***SE of mean*** | **Ca** | ***SE of mean*** |
| Phosphorylated | 1.28E-16 | *1.83E-17* | 6.17E-16 | *2.31E-17* |
| Dephosphorylated | 2.15E-16 | *2.51E-17* | 5.33E-16 | *2.60E-17* |
| Mean Energy Normalised | | | | |
|  | **EDTA** | ***SE of mean*** | **Ca** | ***SE of mean*** |
| Phosphorylated | 1.00E+00 | 1.43E-01 | 4.81E+00 | 1.43E-01 |
| Dephosphorylated | 1.00E+00 | 1.17E-01 | 2.48E+00 | 1.17E-01 |

Supplementary File 1: Descriptive statistics of the adhesive properties of native (phosphorylated) and dephosphorylated OPN film on mica in EDTA and Ca^2+^ ion solutions. The mean energy dissipation in Ca^2+^ buffer was normalized to mean energy dissipation of EDTA buffer.
