# Supplementary material for: The role of extracellular matrix phosphorylation on energy dissipation in bone
Source: eLife. 2020 Dec 9;9:e58184. doi: 10.7554/eLife.58184 (PMC7746230; doi:10.7554/eLife.58184)
Supplement: Supplementary file 2. [file elife-58184-supp2.docx]

| ***pH 8.5*** |  | **OPN on HA** | |  |
| --- | --- | --- | --- | --- |
|  | **Mean** | **STD** | **SE of mean** | **Median** |
| H2O | 1.43E-16 | 7.54E-17 | 1.02E-17 | 1.19E-16 |
| Na | 1.52E-16 | 5.17E-17 | 7.10E-18 | 1.52E-16 |
| Ca | 2.29E-16 | 1.11E-16 | 1.49E-17 | 2.09E-16 |
| *pH 8.5* |  | OPN on HAp Normalised | |  |
|  | **Mean** | **STD** | **SE of mean** | **Median** |
| H2O | 1.00E+00 | 5.25E-01 | 7.08E-02 | 1.00E+00 |
| Na | 1.06E+00 | 3.60E-01 | 4.95E-02 | 1.27E+00 |
| Ca | 1.60E+00 | 7.71E-01 | 1.04E-01 | 1.75E+00 |
|  |  |  |  |  |
| ***pH 6*** |  | **OPN on HA** | |  |
|  | **Mean** | **STD** | **SE of mean** | **Median** |
| H2O | 5.76E-16 | 1.74E-16 | 2.63E-17 | 5.71E-16 |
| Na | 6.46E-16 | 1.27E-16 | 1.78E-17 | 6.52E-16 |
| Ca | 4.02E-16 | 9.45E-17 | 1.30E-17 | 4.01E-16 |
| *pH 6* |  | OPN on HAp Normalised | |  |
|  | **Mean** | **STD** | **SE of mean** | **Median** |
| H2O | 1.00E+00 | 3.03E-01 | 4.57E-02 | 1.00E+00 |
| Na | 1.12E+00 | 2.20E-01 | 3.09E-02 | 1.14E+00 |
| Ca | 6.99E-01 | 1.64E-01 | 2.26E-02 | 7.02E-01 |

Supplementary File 2: Descriptive statistics of the adhesive properties of native (phosphorylated) OPN film on HA under various pH and ionic conditions. The mean energy dissipation in Ca^2+^ and Na^+^ buffers were normalized to mean energy dissipation of H_2_O buffer.
