# Supplementary material for: The role of extracellular matrix phosphorylation on energy dissipation in bone
Source: eLife. 2020 Dec 9;9:e58184. doi: 10.7554/eLife.58184 (PMC7746230; doi:10.7554/eLife.58184)
Supplement: Supplementary file 3. [file elife-58184-supp3.docx]

|  | Mean ForceMax pH6 | | Mean ForceMax pH8.5 | |
| --- | --- | --- | --- | --- |
|  | **Mean** | ***SE of mean*** | **Mean** | ***SE of mean*** |
| **H2O** | 2.56E-09 | *6.28E-11* | 1.96E-09 | *1.12E-10* |
| **Na** | 2.47E-09 | *5.34E-11* | 1.38E-09 | *4.73E-11* |
| **Ca** | 2.49E-09 | *5.66E-11* | 1.36E-09 | *5.57E-11* |

Supplementary File 3: Descriptive statistics of mean maximum force of native (phosphorylated) OPN film on HA under various pH and ionic conditions.
