# Supplementary material for: The role of extracellular matrix phosphorylation on energy dissipation in bone
Source: eLife. 2020 Dec 9;9:e58184. doi: 10.7554/eLife.58184 (PMC7746230; doi:10.7554/eLife.58184)
Supplement: Supplementary file 4. [file elife-58184-supp4.docx]

| Mean “Maximum Force” | | | | |
| --- | --- | --- | --- | --- |
|  | **EDTA** | *SE of mean* | **Ca** | *SE of mean* |
| **Phos** | 1.13E-09 | *9.81E-11* | 7.81E-09 | *1.91E-10* |
| **DePhos** | 1.51E-09 | *2.06E-10* | 8.64E-09 | *3.50E-10* |

Supplementary File 4: Descriptive statistics of mean maximum force of native (phosphorylated) and dephosphorylated OPN film on mica in EDTA and Ca^2+^ ion solutions.
